# The Fungus HL-29: A Promising Weed Pathogen with Bioherbicidal Potential and Crop Safety

**DOI:** 10.3390/jof12010017

**Published:** 2025-12-25

**Authors:** Lan Yang, Chao Peng, Haixia Zhu, Yongqiang Ma

**Affiliations:** 1 Academy of Agriculture and Forestry Sciences, Qinghai University, Xining 810016, China; 15293191375@163.com (L.Y.); pengchao0201@163.com (C.P.); mayongqiang_163@163.com (Y.M.); 2 Qinghai Key Laboratory of Agricultural Integrated Pest Management, Xining 810016, China; 3 Scientific Observation Station of Crop Pests in Xining, Ministry of Agriculture and Rural Affairs, Xining 810016, China

**Keywords:** weeds, herbicidal activity, *Fusarium acuminatum*, crop safety

## Abstract

The herbicidal efficacy and crop safety of Fusarium acuminatum strain HL-29, an endophytic fungus isolated from infected Amaranthus retroflexus in Qinghai Province, were evaluated. In vitro leaf assays demonstrated its pathogenicity against four broadleaf weeds, with efficacy ranked as follows: *Elsholtzia densa* = *Senecio vulgaris* = *Chenopodium album* > *Malva verticillata*. Pot trials further confirmed that the HL-29 fermentate caused 100% disease incidence in *S. vulgaris*, *C. album*, and *E. densa*. Notably, the strain showed no pathogenicity toward seven major local crops, indicating excellent selectivity. Scanning electron microscopy (SEM) revealed key morphological changes during the infection process on *C. album* leaves. These results establish F. acuminatum HL-29 as a promising biocontrol candidate against broadleaf weeds in the Qinghai–Tibet Plateau, providing a theoretical foundation for developing alpine-adapted mycoherbicides.

## 1. Introduction

Weeds represent the most detrimental biotic constraint to crop production, surpassing the combined impact of birds, animals, insects, and pathogens in threatening agricultural yields [1]. Their proliferation poses a major challenge to global farming systems. While effective, the extensive reliance on conventional chemical herbicides has led to significant environmental and agronomic concerns, including pollution, residual toxicity, and the evolution of resistant weed biotypes [2]. In particular, the use of phytopathogenic fungi for weed control has become an active area of research. Plant pathogenic fungi produce bioactive substances that disrupt normal weed growth through the induction of disease. Host-specific fungi have been utilized in the development of bioherbicides [3]. Beyond direct pathogenesis, they often produce phytotoxic metabolites or ethyl acetate extracts with activity against a broader spectrum of weeds, playing complex roles in host physiology and interference [4]. Developing such mycoherbicides aligns with national strategies promoting “ecological civilization” and “green development,” offering a path to reduce chemical dependency [5].

Given these challenges, there is an urgent need to develop efficient and environmentally sustainable microbial weed control technologies. The strategy of “using pathogens to control weeds” offers notable advantages, including high environmental compatibility and a low risk of resistance evolution. A landmark success in this field is the fungus *Phytophthora palmivora*, which was developed to control *Morrenia odorata* in citrus orchards and became the first registered microbial herbicide. In 1981, it became the first microbial herbicide registered by the U.S. Environmental Protection Agency (EPA). (U.S. EPA, 1981). It acts by infecting weed roots, causing rot and plant death [6]. Substantial progress has since been made. Genome-mining approaches have identified novel bioactive compounds such as macrooxamycin A and its derivative MgAG from endophytic Streptomyces bacteria, with MgAG exhibiting a novel mode of action through the inhibition of plant histone deacetylases [7]. From a mycological perspective, several fungi show particular promise. For instance, *Trichoderma* species exhibit broad herbicidal activity against dicot weeds, while various *Fusarium* species demonstrate specific pathogenicity. Notably, crude extracts from *Fusarium* have been shown to induce clear phytotoxicity in water hyacinth [8,9,10], highlighting the significant potential of fungal metabolites in weed biocontrol.

Fungi of the genus *Fusarium* are widely distributed filamentous fungi and represent an important source of bioherbicides [11]. Certain species have demonstrated notable weed suppression; for instance, specific *Fusarium* strains can cause mortality in the toxic weed *Eclipta prostrata* within 13–14 days [12]. The considerable genetic diversity and frequent host specificity observed within this genus provide a sound basis for selecting effective and safe strains for targeted weed control [13]. However, despite confirmed herbicidal potential, research on F.-based bioherbicides remains limited. Most studies have focused primarily on initial activity screening, lacking systematic evaluation of critical factors such as host range specificity and crop safety [14]. This gap is particularly evident in unique agroecosystems like the Qinghai Plateau, where the herbicidal potential of indigenous *Fusarium* strains is poorly explored, and their safety profiles for locally dominant crops have not been comprehensively assessed [15]. This knowledge gap hinders the targeted development and practical application of *Fusarium*-based microbial herbicides in such regions. The present study, therefore, aimed to screen indigenous, highly pathogenic fungal strains from Qinghai to develop bioherbicides targeting four dominant weed species on the Qinghai–Tibet Plateau. Our goal was to provide a novel approach for green weed control and agroecological system protection, specifically tailored for plateau regions. Through in vitro leaf inoculation and pot spray inoculation assays, a systematic evaluation was conducted to assess the pathogenicity of the selected strain against four dominant broadleaf weeds (*M. verticillata*, *S. vulgaris*, *C. album*, and *E. densa*) and its safety to nine major crops in Qinghai. The strain was identified using a combination of morphological and molecular biological methods. The study aims to clarify the herbicidal activity and safety of the strain HL-29. The results will provide an important theoretical basis and microbial resources for developing a novel bioherbicide based on *F. acuminatum*, which has significant implications for advancing green weed control technologies in China.

## 2. Materials and Methods

### 2.1. Sample Collection

**Figure 1:**
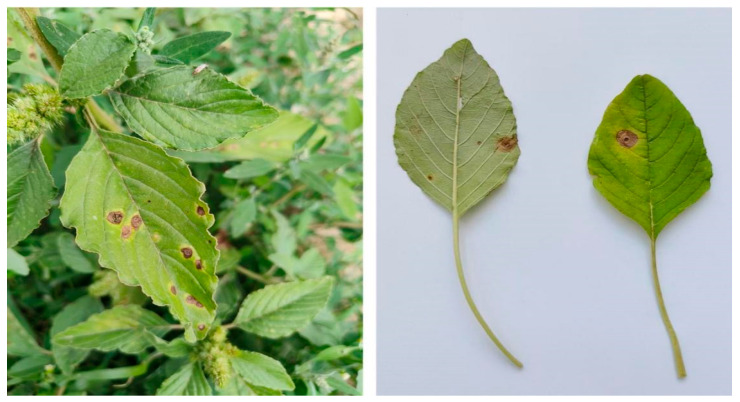
Field habitat (**left**) and symptoms of infected leaves of *A*. *retroflexus* (**right**).

In August 2024, leaves of *A. retroflexus* exhibiting typical disease symptoms (circular brown lesions with distinct margins and yellowish halos) were collected (Figure 1). in Tuanyi Village, Guancang Town, Hualong County, Haidong City, Qinghai Province, along National Highway G213 (36.0300° N, 102.0080° E). Tissue segments were excised from the lesion borders, surface-sterilized, and stored at 4 °C for subsequent fungal isolation.

### 2.2. Isolation and Purification of Fungal Strains

Naturally infected leaves of *A. retroflexus* were gently rinsed with sterile water. Tissue segments (5 mm × 5 mm) were excised from the lesion borders (the junction of diseased and healthy tissue). Surface sterilization was performed by immersing the tissues in 3–5% NaClO solution for 5–15 min, followed by treatment with 70% ethanol for 30 s. After each step, the tissues were rinsed three times with sterile water. The surface-dried segments were then transferred onto Potato Dextrose Agar (PDA) plates and incubated at 28 °C for 5 days. Hyphal tips from the outermost margin of resulting colonies were selected and subcultured to obtain pure isolates.

### 2.3. Strain Identification and Phylogenetic Analysis

#### 2.3.1. Morphological Identification

Purified fungal isolates were inoculated onto Potato Dextrose Agar (PDA) and cultured at 28 °C for 7 days. The morphological characteristics of spores and hyphae were examined microscopically under a microscope. Based on the *Handbook of Fungal Identification* and combined with molecular phylogenetic analysis, the taxonomic status of the strain was systematically determined.

#### 2.3.2. Molecular Biology and Phylogenetic Analysis

Genomic DNA was extracted from the mycelia of a 7-day-old culture using the commercial kit name and brand. The internal transcribed spacer (ITS) region, along with partial sequences of the translation elongation factor 1-alpha (TEF-1α) and RNA polymerase II second largest subunit (RPB2) genes, were amplified via polymerase chain reaction (PCR).

PCR reactions were performed in a Bio-Rad T100 Thermal Cycler (Bio-Rad Laboratories, Inc., Hercules, CA, USA) with a total volume of 25 µL, containing 12.5 µL of 2 × Taq PCR MasterMix, 1 µL of each primer (10 µM), 1 µL of template DNA, and 9.5 µL of nuclease-free water. The thermal cycling conditions were as follows: initial denaturation at 94 °C for 5 min; followed by 35 cycles of denaturation at 94 °C for 30 s, annealing at 55 °C for 30 s, and extension at 72 °C for 1 min; with a final extension at 72 °C for 10 min. To accurately determine the taxonomic identity of the isolates obtained in this study and to reconstruct a robust phylogenetic relationship, a multigene concatenated phylogenetic approach was employed. This method enhances the resolution and statistical support of the phylogenetic tree by combining sequence information from multiple conserved genes. In this study, we targeted three gene loci widely used in fungal phylogenetics: the internal transcribed spacer region of the ribosomal DNA (ITS), the translation elongation factor 1-alpha gene (EF-1α), and the gene encoding the second largest subunit of RNA polymerase II (RPB2). The specific primers used were ITS1/ITS4, EFI-F/EFI-R, and RPB2-5F2/RPB2-7cR, respectively. After PCR amplification, sequencing, and sequence editing for each isolate, the aligned sequences of the three genes were concatenated end-to-end to form a supermatrix alignment. Phylogenetic trees were reconstructed from this combined dataset using Maximum Likelihood or Bayesian Inference methods. Based on this multigene phylogeny, coupled with morphological observations, we precisely delineated the species-level classification of the target isolates within their respective genera. Their phylogenetic placements were supported by high bootstrap values, providing strong molecular evidence for their identification. PCR amplification and sequencing of the ITS, RPB2, and TEF-1α gene regions were conducted by Sangon Biotech (Shanghai) Co., Ltd. (Shanghai, China). The primer sequences used for PCR amplification of the ITS, RPB2, and TEF-1α genes are listed in Table 1.

### 2.4. Pathogenicity Assay on Detached Leaves

Healthy leaves of *E. densa*, *S. vulgaris*, *C. album*, and *M. verticillata* were collected from the field. After being washed with ultrapure water, the leaves were placed in Petri dishes lined with moistened filter paper. Mycelial plugs (5 mm diameter) were taken from the margins of a 7-day-old colony grown on PDA at 28 °C using a sterile tip. Each plug was placed with the mycelial side in contact with the center of a leaf. Plugs of sterile PDA medium served as the control. For each plant species, the experiment was conducted with three biological replicates, each consisting of an independent Petri dish containing three leaf segments. The entire assay was performed twice independently to ensure reproducibility. Data represent the mean values derived from the two independent experimental runs. All inoculated leaves were maintained at 27 °C for 7 days, after which disease development was recorded.

### 2.5. Pathogenicity Assay in Pot Culture

Five mycelial plugs were inoculated into 250 mL of potato dextrose broth (PDB) and incubated at 25 °C with shaking at 180 rpm for 7 days. The fungal broth was filtered through sterile gauze to obtain the fermentation filtrate, to which Tween-80 (1–4 drops per 250 mL) was added. Healthy seedlings of *C. album*, *E. densa*, *M. verticillata*, and *S. vulgaris* at the 4–7 leaf stage (the same developmental stage as the crop seedlings) were transplanted into pots (containing a universal seedling substrate soil) and pre-cultured in a greenhouse at 25 ± 1 °C. The seedlings were sprayed with (HL-29) the fungal filtrate (25–30 mL per pot) for three consecutive days. A control group was treated with PDB containing Tween-80. For each weed species, the experiment was carried out with three biological replicates, each comprising an individual pot containing five seedlings. The entire experiment was conducted twice independently to confirm reproducibility. After inoculation, all pots were covered with plastic film to maintain high humidity. Each treatment included three replicates, and disease incidence was assessed 7 days post-inoculation.

Disease incidence (%) = (Number of infected samples/Total number of samples assessed) × 100

### 2.6. Crop Safety Evaluation

Potted seedlings of seven crop species at the 4- to 7-leaf stage were sprayed with the fungal fermentation filtrate (10 mL per pot) using the method described above. Control plants were treated with sterile PDA. Each treatment was replicated three times. After 7 days of incubation at 25 °C, the phytotoxicity severity was assessed using the following rating scale: NS (no symptoms), LS (leaf scattered spots), MS (20–25% leaf area affected, growth inhibited), and SS (>25% leaf area diseased, growth severely suppressed). For each weed species, the experiment included three biological replicates, with each replicate consisting of an independent pot containing five seedlings. The complete experiment was independently conducted twice to ensure reproducibility of the results.

### 2.7. Scanning Electron Microscopy (SEM) Sample Preparation and Observation

Sample preparation for SEM followed the method described by Haixia Zhu [16]. The infection process of the strain on detached leaves of *C. album* was observed using a JSM-6610LV scanning electron microscope (JEOL Ltd., Tokyo, Japan). Ultrastructural characteristics of fungal development on the leaf surface were analyzed.

### 2.8. Data Analysis

Experimental data were statistically analyzed using SPSS 18.0 software. Multiple-line charts were generated with Origin software. (version 2025, OriginLab Corporation, Northampton, MA, USA).

## 3. Results and Analysis

### 3.1. Results of Strain Identification and Phylogenetic Analysis

#### 3.1.1. Morphological Identification

**Figure 2:**
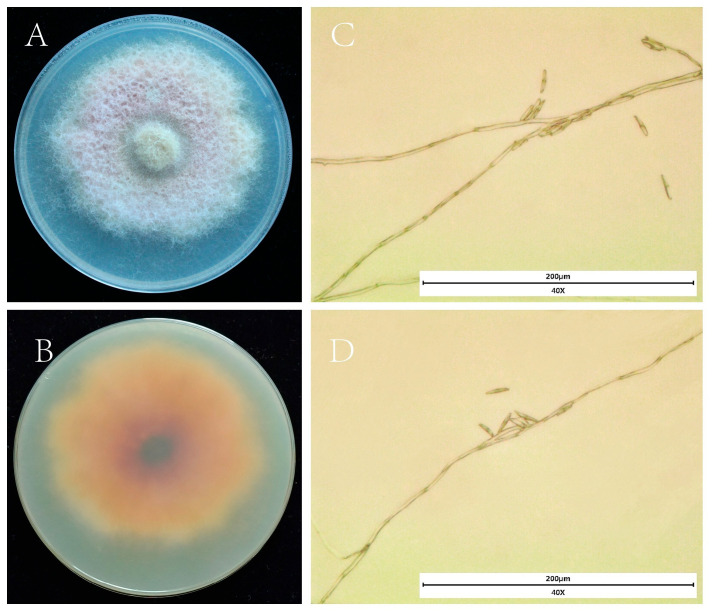
Morphological characteristics of strain HL-29: (**A**,**B**) colony on PDA (front and reverse); (**C**,**D**) conidia and conidiophores.

On PDA medium, the isolate produced colonies with pink mycelia and white margins, exhibiting a raised and fluffy texture. The reverse side of the colony appeared black in the center, with pink radiating streaks on a white background (Figure 2A). The hyphae were transparent, septate, and branched (Figure 2C). Conidia were brown, falcate to lunate, measuring 10–30 μm in length and 1–4 μm in width, with 1–3 septa (Figure 2C). Based on these morphological characteristics, the isolate was consistent with fungi in the genus *Fusarium* and was preliminarily identified as *Fusarium.*

#### 3.1.2. Molecular Identification and Phylogenetic Analysis

**Figure 3:**
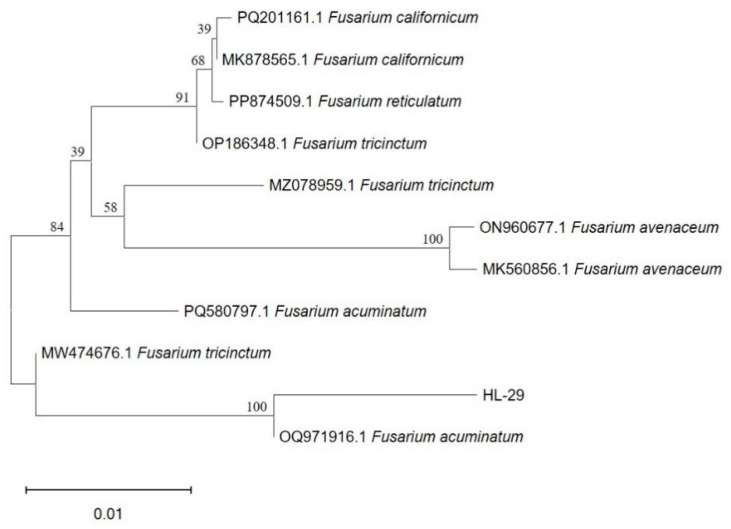
The phylogenetic tree constructed based on sequences of the *ITS*, *GADPH*, and *TEF-1α* genes.

Sequencing results revealed that the ITS region of strain HL-29 was 562 bp in length. Phylogenetic analysis based on the ITS sequence showed that HL-29 exhibited 100% similarity with *F. acuminatum* (accession no. OQ971916.1). Combined with its morphological characteristics and the ITS-based phylogenetic analysis, strain HL-29 was identified as *F. acuminatum* and designated as *F. acuminatum* HL-29 (Figure 3).

### 3.2. Pathogenicity on Detached Leaves

**Figure 4:**
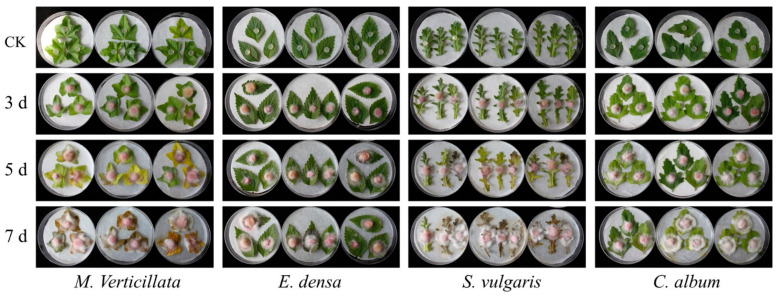
Pathogenicity of strain HL-29 on detached leaves of four weed species.

The pathogenicity of strain HL-29 was evaluated on detached leaves of four weed species. The results demonstrated that HL-29 exhibited notable pathogenic activity against *C. album*, *E. densa*, *M. verticillata*, and *S. vulgaris*. Symptoms observed at 3, 5, and 7 days post-inoculation are shown in Figure 4. *M. verticillata* was the most severely affected, with leaves progressing from chlorosis to dark brown or black discoloration. *S. vulgaris* and *C. album* showed moderate symptoms. Inoculated leaves of *C. album* displayed white mycelial growth and brown necrotic lesions at the inoculation sites, with central yellowing. Leaves of *S. vulgaris* also exhibited yellowing to dark brown lesions. Although vigorous white mycelial growth was observed on leaves of *E. densa*, penetrating the entire leaf tissue and accompanied by yellowing, the overall tissue damage was the mildest among the tested species. Based on the severity of symptoms, the pathogenicity of HL-29 against the four weeds was ranked as follows: *M. verticillata* > *S. vulgaris* > *C. album* > *E. densa.*

### 3.3. Pathogenicity in Pot Culture

**Figure 5:**
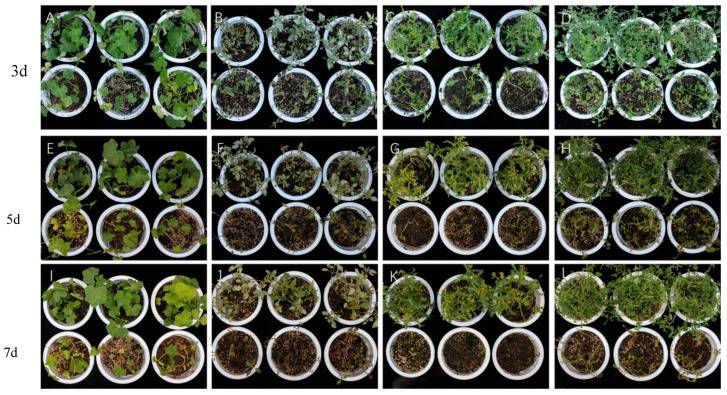
Pathogenicity of strain HL-29 on four weed species at 3, 5, and 7 days post-inoculation: (**A**,**E**,**I**) symptoms on *M. verticillata* at 3, 5, and 7 dpi, respectively; (**B**,**F**,**J**) symptoms on *E. densa* at 3, 5, and 7 dpi, respectively; (**C**,**G**,**K**) symptoms on *S. vulgaris* at 3, 5, and 7 dpi, respectively; (**D**,**H**,**L**) symptoms on *C. album* at 3, 5, and 7 dpi, respectively. Note: In each image panel, the upper row represents the control group, and the lower row represents the treated group.

**Figure 6:**
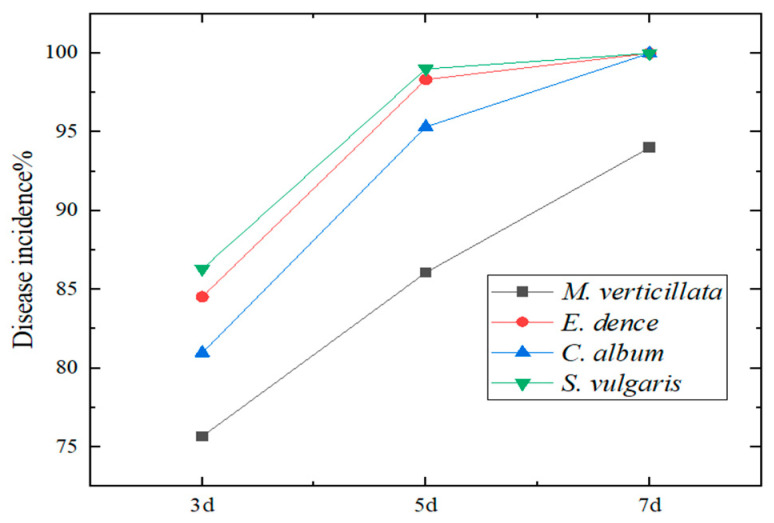
Disease incidence in pot culture at 3, 5, and 7 days post-inoculation.

Initial disease symptoms were observed on all four target weed species at 3 days post-inoculation, including localized chlorosis, leaf and stem wilting, and reduced leaf size (Figure 5 and Figure 6). By 5 days post-inoculation, disease incidence rapidly increased to over 90% in *E. densa*, *S. vulgaris*, and *C. album*, while reaching 85% in *M. verticillata*. The relatively lower incidence observed in *M. verticillata* was speculated to be associated with its deeper root system. At this stage, symptomatic plants showed water-soaked stems. At 7 days post-inoculation, the final disease incidence reached 94% for *M. verticillata*, and 100% for each of *E. densa*, *S. vulgaris*, and *C. album*. Almost all plants of the latter three species were completely necrotic, while only a very limited number of *M. verticillata* plants retained a small amount of healthy tissue.

### 3.4. Safety Evaluation Results for Crops

**Figure 7:**
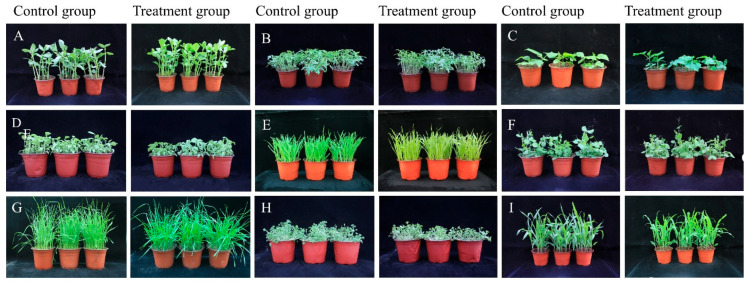
Effect of strain HL-29 spore suspension on crop safety: (**A**) *Vicia faba* “Qinghai 9” from control (**left**) and treatment (**right**), same as below; (**B**) *Solanum lycopersicum* “Boyu 368”; (**C**) *Cucumis sativus* “Zhongnong 5”; (**D**) *Solanum melongena* “Huaqie1”; (**E**) *Hordeum vulgare* “Kunlun 18”; (**F**) *Pisum sativum* “Caoyuan 224”; (**G**) *Triticum aestivum* “Qingchun 38”; (**H**) *Brassica napus* “Qingza 5”; (**I**) *Zea mays* Zhongnongdaqingzhu 67”.

At 7 days post-inoculation, the disease development in crops was investigated. As shown in (Figure 7) and (Table 2), no symptoms were observed in faba bean, tomato, cucumber, eggplant, pea, oilseed rape, or maize compared to the control plants. These crops exhibited normal growth and leaf color without any lesions, and were assigned a safety rating of NS. In contrast, wheat and hulless barley showed slight growth inhibition with minor leaf yellowing, indicating mild phytotoxicity, and were rated as LS.

### 3.5. Scanning Electron Microscopy Observation of Ultrastructural Features During Infection of C. album by HL-29

**Figure 8:**
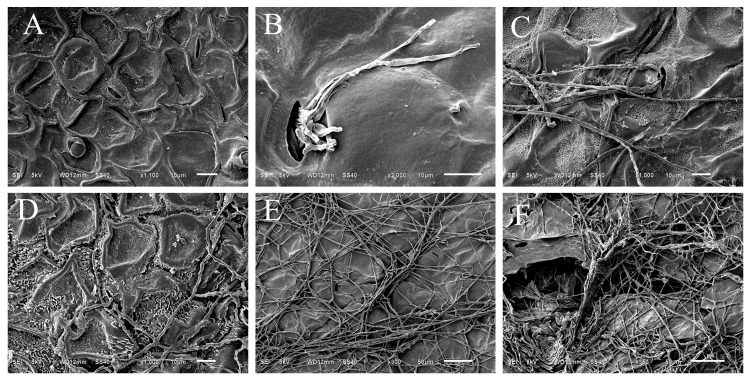
Scanning electron microscopy (SEM) observation of the ultrastructural changes in *C. album* infected by strain *HL-29*: (**A**) infection status at 0 days post-inoculation with HL-29; (**B**) infection status at 3 days; (**C**,**D**) infection status at 5 days; (**E**,**F**) infection status at 7days.

As shown in Figure 8, the surface of non-inoculated hyphae remained intact (Figure 8A). At 2 days after inoculation, hyphae were observed penetrating through the stomata, while the leaf tissue structure remained unaffected (Figure 8B). Within 2 days, multiple hyphae had colonized the area surrounding the stomata (Figure 8C). Within 3 to 4 days, hyphal growth became vigorous, forming a dense hyphal network (Figure 8D,E), accompanied by minor damage to the leaf tissues. At 5 days, clear symptoms of tissue disruption were evident on the surface. Hyphae and spores were parasitic on the tissue surface, absorbing nutrients, and the plants exhibited pronounced disease symptoms (Figure 8F).

## 4. Discussion and Results

This study confirms that *F. acuminatum* strain HL-29, isolated from infected *A. retroflexus* in Qinghai Province, exhibits strong herbicidal activity against key weed species. Under both laboratory and greenhouse conditions, the strain demonstrated high pathogenicity toward *E. densa*, *S. vulgaris*, and *C. album*, and also induced yellowing and mortality in *M. verticillata*. These results underscore the significant potential of *F. acuminatum* HL-29 as a biocontrol agent against several prevalent weeds on the Qinghai–Tibet Plateau. The findings align with and extend previous reports on the role of *Fusarium* species as promising fungal agents for weed management [17].

In vitro leaf and pot experiments demonstrated that *F. acuminatum* infection resulted in leaf yellowing, wilting, root damage, and inhibition of seed germination. These symptoms are consistent with the production of toxins typically associated with the pathogenic mechanisms of *Fusarium* species [18]. Previous studies have indicated that *F. acuminatum* exhibits strong pathogenic ability, colonizing the vascular system after infection and disrupting xylem function, ultimately leading to host plant death [19,20,21,22]. During the infection process, this fungus induces multiple virulence factors that damage host cells [23] and releases various toxins that contribute to disease development and weed mortality [24,25]. However, the production of such mycotoxins, particularly DON, also raises important biosafety considerations for its potential use as a bioherbicide. Among these, the toxin deoxynivalenol (DON) has been shown to inhibit normal seed germination, lateral root formation, and the growth of shoots and callus [26], representing a unique mechanism for weed biocontrol. If the fungus were to be applied in the field, the potential for toxin persistence in soil or translocation to non-target plants must be rigorously evaluated to ensure no adverse effects on subsequent crops, food safety, or the environment [27,28]. According to Walker et al., *F. acuminatum* can be transmitted via seeds and is pathogenic to certain ferns, causing pre- and post-emergence damping-off, root rot, and hypocotyl necrosis [29]. This underscores the need for targeted application strategies to minimize off-target effects. Furthermore, scanning electron microscopy observations revealed that hyphae penetrate the leaf surface of *C. album* primarily through stomatal openings, impairing normal plant growth. This suggests that the strain likely employs a similar pathogenic strategy: disrupting xylem function and inducing or releasing virulence-related toxins, ultimately leading to weed death. Similar to other fungal agents used in weed biocontrol, *F. acuminatum* shows promise as a potential bioherbicide. For its safe deployment, future research should prioritize quantifying toxin production under field conditions and assessing the environmental fate of these compounds to develop effective risk mitigation protocols [30].

Crop safety assessment revealed that the evaluated results were mostly classified as NS (non-sensitive), with only barley and wheat showing LS (low sensitivity). In fact, the strain demonstrated safety toward legumes, crucifers, cucurbits, and solanaceous crops. This finding is of critical importance, as it indicates a highly restricted host range of the strain, providing a strong safety basis for agricultural applications. Its non-pathogenicity to these crops ensures that it poses no threat to major rotation system components during field use, significantly reducing the risk of damage to non-target plants—a prerequisite for any bioherbicide candidate. Notably, there have been no prior reports, domestically or internationally, on the use of *F. acuminatum* as a bioherbicide. This strain thus holds considerable potential for development as a host-specific microbial herbicide. Although slight sensitivity was observed in barley and wheat, the symptoms did not include plant death or substantial economic yield loss, which fundamentally distinguishes them from wilting or rot diseases. In contrast to many conventional herbicides, it poses a lower environmental risk due to the absence of synthetic chemical residues. Such minor sensitivity can be effectively mitigated through appropriate application techniques, such as avoiding sensitive growth stages of barley and wheat and employing precise inter-row directional spraying to prevent contact with crop leaves. Importantly, this study is the first to identify *F. acuminatum* as a potential biocontrol agent against weeds of the Qinghai–Tibet Plateau. This absence of prior reports underscores the innovative nature of our work. Compared with other reported biocontrol strains, *F. acuminatum* HL-29 stands out due to its safety to most crops and high efficacy against target weeds. This high selectivity constitutes a core competitive advantage for its successful transition into practical application. To translate this promising discovery into a commercially viable product, future efforts must address key challenges, including scaling up fermentation processes, optimizing formulation for field stability and efficacy, and navigating the regulatory pathway for microbial herbicide registration. Furthermore, a major challenge for the successful field application of any bioherbicide is its performance under fluctuating environmental conditions, such as variable temperature, humidity, and UV radiation, which can significantly impact the survival, germination, and infectivity of fungal propagules [31,32]. While our laboratory assays were conducted under optimal conditions (25 °C and high humidity), future research must focus on formulating the product and defining application windows that maximize efficacy under the specific climatic conditions of the target regions.

The high safety profile of this strain may be intrinsically linked to its pathogenic mechanism. Previous studies have indicated that the pathogenicity of *Fusarium* species is often closely associated with the production of specific toxins, such as trichothecenes. We speculate that this strain may naturally lack key genes required for the synthesis of certain toxins, or that its toxin biosynthesis pathways are tightly regulated. As a result, it can only infect target weeds possessing specific susceptibility mechanisms, while remaining largely non-pathogenic to most crops. This hypothesis provides a clear research direction for future molecular-level analysis of its host specificity, and even for further disruption of toxin synthesis genes via genetic engineering to completely eliminate any potential risk to crops.

In summary, the strain *F. acuminatum* HL-29 demonstrates outstanding safety—characterized by high non-pathogenicity toward multiple major crops and limited, manageable effects on graminaceous species—coupled with its current scarcity in international research. These attributes establish its strong potential as a candidate bioherbicide for weed control. Subsequent research will focus on optimizing application strategies to mitigate potential impacts on wheat and barley, and on elucidating the molecular basis of its host specificity. The endophytic fungal strain *HL-29*, isolated from diseased *A. retroflexus* leaves collected in Tongjie Village, Qunke Town, Hualong Hui Autonomous County, Haidong City, Qinghai Province (along National Highway G213), was selected to evaluate its herbicidal activity and safety toward major crops in Qinghai. Preliminary screening using in vitro leaf inoculation revealed that the pathogenicity of strain HL-29 against four common broadleaf weeds followed the order *E. densa* = *S. vulgaris* = *C. album* > *M. verticillata*. To further verify its herbicidal efficacy, in vivo plant spray inoculation was conducted under pot conditions. The fermentation broth of HL-29 exhibited high pathogenicity toward *S. vulgaris*, *C. album*, and *E. densa*, with disease incidence reaching 100% in all cases. Meanwhile, the strain showed no significant pathogenicity toward major crops in Qinghai, including broad bean, pea, maize, rapeseed, cucumber, eggplant, and tomato, indicating favorable biosafety toward these crops. However, strain HL-29 inhibited plant height and growth vigor and induced noticeable disease symptoms in barley and wheat, suggesting potential risks in fields where these crops are cultivated. Based on combined morphological and molecular identification, strain HL-29 was identified as *F. acuminatum*. Overall, HL-29 exhibits strong pathogenicity against multiple broadleaf weeds and highly selective safety toward most major crops, highlighting its potential for development as a microbial herbicide. However, before practical agricultural application, further assessment is required on key issues such as environmental adaptability, stability of field control efficacy, and effects on non-target organisms.

## Figures and Tables

**Table 1:** Primer sequences used for PCR amplification of the ITS, RPB2, and TEF-1α genes.

Primer	Nucleotide Sequence
ITS1	5′-TCCGTAGGTGAACCTGCGG-3′
ITS4-R	5′-TCCTCCGCTTATTGATATGC-3′
EFI-F	5′- CATCGAGAAGTTCGAGAAGG-3′
EFI-R	5′-TACTTGAAGGAACCCTTACC-3′
RPB2-5F2	5′-GGGGWGAYCAGAAGAAGGC-3’
RPB2-7cR	5’-CCCATRGCTTGYTTRCCCAT-3’

**Table 2:** Disease assessment of HL-29 on crops.

Test Crops	Disease
*T. aestivum*	LS
*P. sativum*	NS
*H. vulgare*	LS
*V. faba*	NS
*B. napus*	NS
*C. sativus*	NS
*Z. mays*	NS
*S. melongena*	NS
*S. lycopersicum*	NS

## Data Availability

The original contributions presented in this study are included in the article. Further inquiries can be directed to the corresponding author.
